# Measurement of Electron-Ion Relaxation in Warm Dense Copper

**DOI:** 10.1038/srep18843

**Published:** 2016-01-06

**Authors:** B. I. Cho, T. Ogitsu, K. Engelhorn, A. A. Correa, Y. Ping, J. W. Lee, L. J. Bae, D. Prendergast, R. W. Falcone, P. A. Heimann

**Affiliations:** 1 Center for Relativistic Laser Science, Institute for Basic Science (IBS), Gwangju, 500-712, Korea; 2 Department of Physics and Photon Science, Gwangju Institute of Science and Technology (GIST), Gwangju, 500-712, Korea; 3 Lawrence Livermore National Laboratory, Livermore, CA 94550, USA; 4 Advanced Light Source, Lawrence Berkeley National Laboratory, Berkeley, CA 94720, USA; 5 Molecular Foundry, Lawrence Berkeley National Laboratory, Berkeley, California 94720, USA; 6 Department of Physics, University of California, Berkeley, CA 94720, USA; 7 Linac Coherent Light Source, SLAC National Accelerator Laboratory, Menlo Park, CA 94025, USA

## Abstract

Experimental investigation of electron-ion coupling and electron heat capacity of copper in warm and dense states are presented. From time-resolved x-ray absorption spectroscopy, the temporal evolution of electron temperature is obtained for non-equilibrium warm dense copper heated by an intense femtosecond laser pulse. Electron heat capacity and electron-ion coupling are inferred from the initial electron temperature and its decrease over 10 ps. Data are compared with various theoretical models.

Under extreme temperature and pressure conditions, normal matters behave abnormally and often regulates physical processes in the stars and large planets^1^, inertial confinement fusion^2^ as well as the applied processes of laser machining and ablation^3^. As an example of such extreme conditions, the warm dense matter (WDM) regime represents the state between condensed matter and plasma, where the thermal energy is comparable to the Fermi energy and the ions are strongly coupled^4^. As an intermediate state of matter between the well-defined disciplines of condensed matter and plasmas, investigation of the WDM regime provides key understanding of non-equilibrium phase transitions and energy relaxation processes in the extreme conditions. However, the complicated interplay of the physical processes in WDM creates difficulty for theoretical description. In addition, the extremely high temperature and pressure conditions associated with WDM create significant challenges for detailed experimental studies^5^.

Most experimental methods for generating WDM put energy into either the electrons or ions. The material then evolves toward equilibrium. Femtosecond laser excitation or laser generated proton beam heating of thin metal foils are widely used techniques^6^,^7^,^8^,^9^,^10^,^11^,^12^,^13^,^14^. Initially, a strongly non-equilibrium state is created and then energy is transferred from the hot electrons to the cold lattice. However, the fast relaxation dynamics (typically on a ps time scale) makes the measurement of WDM thermophysical properties, such as heat capacity and electron-ion coupling, extremely difficult. Instead, well-known thermodynamic data or theoretical descriptions based on either plasmas or solids have often been adopted without rigorous experimental validation^6^,^7^,^8^.

For strongly heated fluid / plasma systems, the energy relaxation processes are often modeled through direct two-particle scattering or ion acoustic modes^15^,^16^. Generally, the relaxation times estimated by such models taking into account the collective behavior of coupled modes are much longer than early predictions^17^,^18^, and strong indications of such longer relaxation times are found in some experiments^19^,^20^. On the other hand, Lin *et al.* presented the computational study of the temperature - dependent thermophysical properties of various metals irradiated by an intense femtosecond laser pulse^21^,^22^. In this work, it was included a high temperature Fermi distribution and electron density-of-states (DOS) to calculate electron heat capacities, the electron-phonon couplings and chemical potentials of high-temperature metals showed large deviations from the widely used free-electron models^21^. A series of investigations have used Lin *et al.’*s calculations to interpret experimental data for optical conductivity and electron diffraction in WDM regime^10^,^12^,^14^. However, applying these results requires caution because they assume the DOS of solid-state materials and the room temperature phonon spectra. The consideration of non-equilibrium electron distributions is also lacking. Therefore, direct measurements of thermodynamic properties in the WDM regime are required.

In this paper, we present an experimental investigation of the electron heat capacity and the electron-ion relaxation of copper in warm and dense conditions. With the strong femtosecond optical excitation of a tamped copper nano foil, the initial non-equilibrium warm dense copper (WDCu) states are created and then advance toward equilibrium via energy exchange between the two sub-systems at a constant density. Utilizing picosecond x-ray absorption spectroscopy, the temporal evolution of electron temperatures of WDCu is determined. From the initial value and the decrease of the electron temperature, the electron heat capacity and the electron-ion coupling are inferred. The experimental results are compared with Lin’s model as well as with new calculations using the high-temperature liquid DOS.

## Experimental Setup

**Figure 1:**
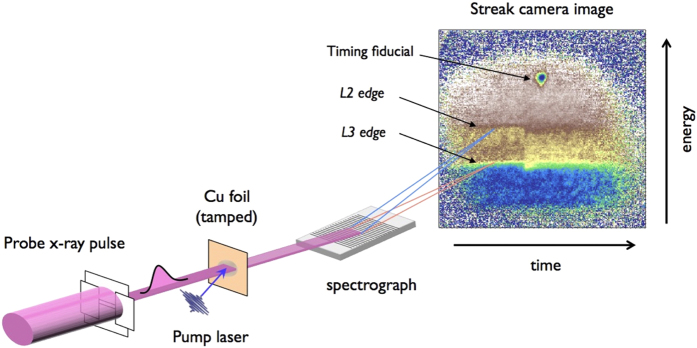
Schematic of the experimental set-up. The femtosecond optical pulse is focused on the sample to provide 0.32 ~ 0.6 J/cm^2^ absorption fluence. The broadband x-ray beam (900 ~ 1,000 eV) from the ALS undulator is overlapped with the optical laser pulse on the sample.

For the experiment performed at the beamline 6.0.2 of the Advanced Light Source (ALS), we employed the femtosecond laser pump - picosecond x-ray probe technique [Fig. 1]. Details of setup are described in the references^23^. The sample is a 70 nm thick foil of copper coated with 100 nm silicon-dioxide layers on both sides. Reflection and transmission measurements of the Ti:sapphire laser pulse (800 nm, 150 fs) in the experimental geometry indicate that 80 ~ 95% of incident laser energies are transmitted through the silicon-dioxide layer, and the absorbed laser fluences to copper sample are estimated to be 0.32 ~ 0.60 J/cm^2^ depending on the pulse energies. Since the femtosecond pulse heats the sample isochorically, corresponding energy densities *E*_*d*_ are 3.5 ~ 6.5 × 10^6^ J/kg.

Under these conditions, the temperature of the sample will be of the order of an electron volt (eV), which is higher than the melting and boiling points. The high temperature fluid will undergo expansion dynamics and the expansion speed of the surfaces of bare sample is estimated to be an order of sound speed (~10^3^ m/s). This hydromotion poses a challenge to the experiment that requires a well-defined density. Previous experiment showed that after ~10 ps, isochoric conditions no longer hold and expansion may affect the sample temperature^12^. Therefore, the sample is “tamped” in order to delay the expansion of the foil^24^. The tamping silicon dioxide layers have a large band gap and prevent the copper foil from expanding on a time scale *2d*/*v* ~ 30 ps, where *d* = 100 nm is the thickness of the tamping layer and *v* ~ 6000 m/s is the sound speed in SiO_2_ at ambient conditions. This delay of the expansion allows us to observe the equilibration dynamics of warm dense copper at a constant volume and density.

X-ray Absorption Near Edge Spectroscopy (XANES) at the copper *L* edge is performed using broadband 70 ps x-ray pulses from an undulator source. Transmitted x-rays are dispersed by a spectrometer and detected by an x-ray streak camera. Spectral and temporal resolutions are 1 eV and 2 ps, respectively. The streak camera records a series of XANES spectra during the x-ray pulse duration.

## Results

### TR-XANES and electron temperature measurement

**Figure 2:**
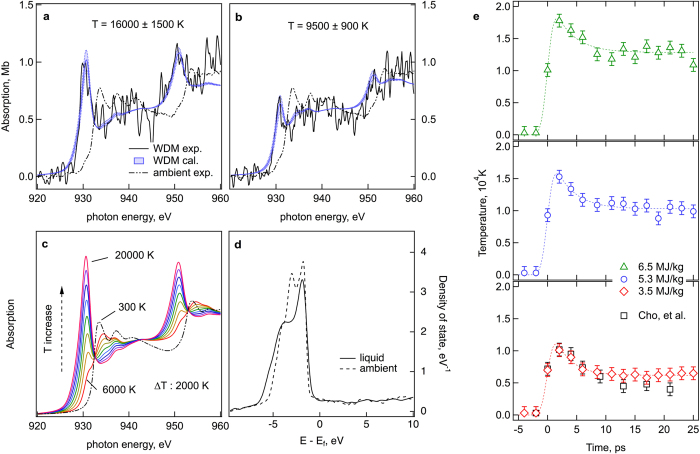
XANES and electron temperature. Determination of electron temperature of WDCu by comparison of the experimental XANES (black solid curve) and calculations (blue). 6.5 MJ/kg at 2 ps (**a**) and 3.5 MJ/kg at 4 ps (**b**). For comparison, the ambient Cu spectrum is also shown (double dotted line). (**c**) Calculated warm dense XANES at various electron temperatures. (**d**) High temperature liquid and ambient solid Cu density of states used to calculate the Fermi distribution functions, which in turn, were used to calculate the XANES in c. (**e**) Evolution of electron temperatures in WDCu. Three different laser fluencies are irradiated on 70 nm copper foils. In the two temperature description, electrons and ions are equilibrated after ~10 ps. Final temperatures are approximately 13,000 K, 11,850 K and 6,400 K, respectively.

Figure 2(a,b) exhibit typical time-resolved (TR) XANES spectra measured at different energy densities. For comparison, the ambient spectrum is also presented. The strong red shift of the *L* edge for WDCu is a consequence of the elevated electron temperature. For Cu, the high-energy edge of the 3*d* band is located 2 eV below the Fermi level. Therefore, Fermi distributions of 1 ~ 2 eV electron temperatures can generate significant unoccupied states in the 3*d* band and 2*p* → 3*d* photo-absorption is allowed. The peaks at 930 and 950 eV represent these transitions with different initial angular momentums, 3/2 and 1/2. Note that the pre-edge peaks are very prominent in this work compared to the data in the previous work^12^. In this work, much stronger laser pulses created higher temperature conditions and more unoccupied *d*-states. In addition, an improved spectral resolution allows us to resolve clearly peaks and structures near the absorption edge for both ambient and warm dense spectra.

Using the electron DOS of high temperature liquid copper^12^, warm dense XANES spectra are calculated at various electron temperatures and presented in Fig. 2(c). Details of the calculations are described in the Method section. The series of spectra reproduce the essential changes at the *L* edges observed in the experiment. The higher the electron temperature, the stronger is the absorption seen near 930 and 950 eV. The height and width of these peaks are sensitive to the electron temperature. Hence, with the proper adjustment of electron temperature and corresponding Fermi distribution in the liquid DOS, good agreement between the calculated and experimental XANES spectra can be obtained [Fig. 2(a,b)].

From XANES spectra at different delays, we determine how the electron temperature of warm dense copper evolves at various energy densities [Fig. 2(e)]. Although the initial energy deposition (3.5 ~ 6.5 MJ/kg) happens at *t* = 0, due to the time response of detector, the peak temperatures are always observed at 2 ps, which is the temporal resolution of the experiment. At *t* = 0, the observed temperatures represent a sampling of both the ambient (*t* < 0) and warm dense (*t* > 0) states.

The sample thickness is matched with the electron mean-free-path at the Fermi level (70 nm)^25^. It is considered that the thermal gradient across the thickness is minimal, and the heat transfer scenario is simplified. The decreasing electron temperature is primarily due to the energy transfer from the hot electrons to the initially cold ions. Figure 2(e) shows that for all three energy densities, equilibrium is obtained within a time scale of 10 ps. The importance of isochoric conditions obtained with the tamping layers is seen in the bottom panel of Fig. 2(e) by the comparison with data from the reference^12^. Whilst the initial dynamics up to ~6 ps are similar, at longer time delays, the electron temperature in the previous work decreases further. A temperature of ~4,000 K was reached, which is ~30% lower than in the present experiment. Hydrodynamic expansion might have caused this additional cooling.

### Electron and ion heat capacities

From the data in Fig. 2(e) and the framework of the two-temperature model (TTM), which is widely used to describe the non-equilibrium systems created by femtosecond laser pulses, we attempt to determine the electron heat capacity and electron-ion coupling at high temperatures.

**Figure 3:**
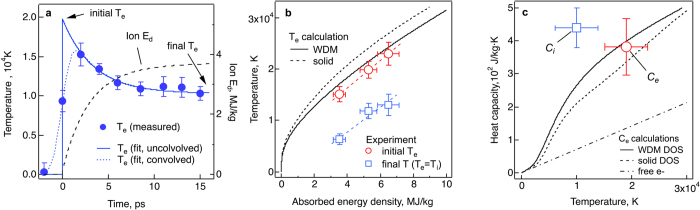
Electron and ion heat capacities. (**a**) Fitting of electron temperatures with a causal exponential function for the energy density 5.3 MJ/kg. (**b**) Initial electron temperatures vs. absorbed energy densities. Experiments (red hexagons) and approximations based on WDM and solid DOS. Final temperatures vs. absorption energy densities (blue squares) are also shown. (**c**) Electron heat capacity at around 20,000 K (red hexagon). Electron heat capacities calculated based on WDM, solid DOS and free electron model. Ion heat capacity vs. temperature (blue square) at around 10,000 K is also determined.

The time scale of the energy deposition by the laser pulse, ~150 fs is much shorter than the time resolution of the streak camera and the time scale of electron – ion equilibration. Thus, we assume the initial energy from a delta-function like laser pulse is deposited into only electrons creating a high temperature Fermi distribution instantly. At this stage, energy deposition to the lattice is not considered. Without a good prior knowledge of energy relaxation in this regime, the electron temperature is considered to decrease exponentially. The temperature data in Fig. 2(e) are fit with a causal exponential function (0, for *t* < 0 and *a* × exp(–*t*/*τ*) + *b,* for *t* > 0) convolved with the detector response. Figure 3(a) shows an example of such a fit for the 5.3 MJ/kg energy density. The initial electron temperature (*a* + *b*), is determined as 19,800 K. For three different energy densities, all fitting results are summarized in the Table 1. The initial electron temperatures for three cases are shown in the Fig. 3(b) [red circle]. For comparison, the initially absorbed energy density in the electron system, *E*_*d*_*(T*_*e*_) = *∫ f(ε,μ,T*_*e*_*)g(ε)εdε*, is calculated using both the WDM and ambient density of states, where *f, g, ε, μ* are the electron distribution, density of states, electron energy, and chemical potential, respectively. It is noted that the WD Cu is in the liquid phase. Overall, with a given *E*_*d*_, the electron temperatures calculated with the WDM DOS are lower than with the ambient DOS and in closer agreement to the experiment.

The theoretical predictions of the temperature dependent electron heat capacity are also presented in the Fig. 3(c). The Sommerfeld expansion *C*_*e*_*(T*_*e*_) = *γT*_*e*_, and *C*_*e*_*(T*_*e*_) = *∫ ∂f/∂T*_*e*_*g(ε)εdε* with solid Cu DOS are from the refs 21,26. In addition, we also calculated *C*_*e*_*(T*_*e*_) with the liquid DOS. Experimentally, it is hard to determine a temperature dependent heat capacity function with the three data points in Fig. 3(b). Instead, we made a linear fit in the temperature range of 15,000–23,000 K and determined *C*_*e*_ at around 20,000 K to be 380 ± 80 J/kg·K. The result is clearly several-fold larger than the linear dependence, *γT*_*e*_, and validates the argument of Lin *et al*. that the details of a material’s electronic structure should be included in a model of the high temperature heat capacity^21^.

The predicted heat capacity based on the liquid DOS provides a good agreement with the experimental data. But within the present uncertainty, the ambient DOS curve cannot be excluded. Further investigations with improved time resolution will be required.

The analysis presented above describes the transient non-equilibrium dynamics and electronic heat capacity in the vicinity of 20,000 K. We note that the thermally equilibrated electron-ion state reached after 10 ps (Fig. 2(e), and the third column of Table 1) can be independently used to determine the total heat capacity in the temperature range around 10,000 K (blue squares in Fig. 3(b)). From a linear fit of equilibrium temperatures and deposited energy densities, we obtain a specific heat of 440 ± 100 J/kg·K at around 10,000 K. A common assumption in equation of state modeling is that the heat capacity is the sum of electronic *C*_*e*_ and ionic *C*_*i*_ contributions. By using the model electronic heat capacity of Lin at 10,000 K as shown in Fig. 3(c), the ionic heat capacity, *C*_*i*_ = 170 ± 100 J/kg·K is obtained. For reference, it corresponds to an ionic heat capacity 1.3 *k*_B_/atom, near ideal gas heat capacity and lower than Dulong-Petit. Due to possible heat loss and expansion in the scale of 10 ps, the obtained value might be an underestimation of the heat capacity. This result, however, can be important to constrain the thermodynamic models of dense liquids in this largely unexplored range of temperatures^27^.

### Electron – ion coupling

The temperature dependent electron-ion coupling is also determined from the electron temperature, *T*_*e*_*(t)* = *a* exp(−*t*/*τ*) + *b* and temperature-dependent electron heat capacity, *C*_*e*_. For the three energy densities, 3.5, 5.3 and 6.5 MJ/kg, the time constants, *τ* are 2.8 ± 0.4, 3.4 ± 0.7 and 3.3 ± 1.0 ps respectively. Placing *T*_*e*_*(t)* into the set of TTM equations, the time-dependent electron-ion coupling can be obtained as followings


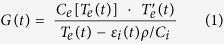


where *ε*_*i*_*(t)* = *∫* *C*_*e*_*[T*_*e*_*(t)]/ρ a/τ exp(−t/τ) dt* and *ρ* are the ion energy density and the mass density respectively.

For the electron temperature, the measured values in the Fig. 2(e) are used. Here, we note that *G* is proportional to the derivative of *T*_*e*_. Within the given experimental accuracy, the small temperature variation after 8 ps is insufficiently determined. Therefore, the electron temperatures and their change at 2, 4 and 6 ps are used. For the electron heat capacity, *C*_*e*_*(T*_*e*_) is required over the temperature range of the cooling sample. In the previous section, we determined *C*_*e*_ near 20,000 K and validated the DOS dependent heat capacity. Here we adopt the calculated *C*_*e*_*(T*_*e*_) based on the WDM DOS.

**Figure 4:**
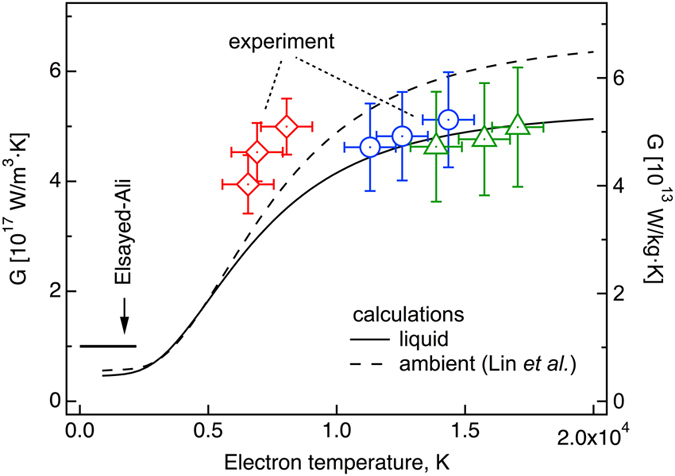
Electron – ion couplings of warm dense copper. (**a**) The *G* obtained by Eq. (1) at 2, 4 and 6 ps for three different energy densities are displayed at the corresponding electron temperatures together with electron-phonon coupling calculations using Eq. (2) with both liquid DOS and ambient solid DOS^21^. For comparison, the electron-phonon coupling constant of solid Cu is also shown^28^.

For three energy densities, the *G* values for electron temperatures at 2, 4 and 6 ps are plotted in the Fig. 4. The electron-ion couplings are strongly dependent on electron temperature in 6,000 ~ 10,000 K range, but relatively unchanged in 10,000 ~ 20,000 K. Overall, they are found to be 4 ~ 6 × 10^17^ W m^−3^ K^−1^, which are a factor of 4 ~ 6 greater than the known electron-phonon coupling of solid copper, *G*_*0*_ = 10^17^ W m^−3^ K^−1^ 28.

We have calculated the electron-ion coupling parameter using the following relation from the ref. 21 with both the ambient and liquid density of states.


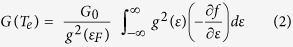


where *G*_*0*_ is 
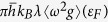
. In this expression, 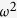 is the second moment of the phonon spectrum^29^ and λ is the electron –phonon mass enhancement parameter^30^. Overall, both curves show a similar trend, that in the range of 5,000* *~ 10,000 K, *G(T*_*e*_) rapidly increases and then varies slowly in >10,000 K.

Compared with the experiment in the high temperature range (>10,000 K), the WDM calculations provide a good agreement with the data (~ 5 × 10^17^ W m^−3^ K^−1^). The ambient DOS predicts about 20% higher coupling than the measurement and WDM DOS. In the lower temperature range (<10,000 K), a strong temperature dependency is well reproduced, but both calculations show lower values than the measurement.

Two points should be noted here. First, the experimental time resolution, 2 ps, is slower than a typical time scale of lattice disordering. Therefore experimental data at 2 ps may include multiple phases *i.e.* solid, liquid and their mixtures. In particular for the case of <10,000 K, the contributions from lower temperature phases would be greater. For the proper description of this complicate regime, accurate knowledge about non-equilibrium melting process would be required. Second, for the Eq. (2), the room temperature lattice and a relevant phonon spectrum are assumed^21^. A good agreement with our interpretation of experimental data using a single exponential fitting may indicate that the coupling between the electrons and low-temperature phonon are still a major pathway of energy exchange even for warm dense condition of copper. Future research with higher time resolution would allow high precision measurements of the electron heat capacity and election-ion coupling at various temperatures.

## Discussion

In this paper, we have presented the experimentally determined electron heat capacity and electron-ion relaxation of warm dense copper heated by an intense femtosecond laser pulse. The evolution of electron temperature was measured using the picosecond x-ray absorption spectroscopy technique. The electron heat capacity and electron-ion relaxation were determined from the initial electron temperatures and their decrements. Comparisons with the thermal properties of ambient copper and various theoretical predictions confirmed that these properties are sensitive to electron temperature and details of electronic structure. Both quantities exhibited the best agreement with the calculations including high temperature electron distributions and the liquid density of state, albeit the ambient DOS could not be excluded. Finally, it is noted that recent x-ray free electron laser (XEFL) based experiments have demonstrated the possibility to study the lattice disorder and ion temperature in warm dense conditions^31^,^32^ and to perform ultrafast XANES measurements^33^,^34^. Combining ultrafast x-ray spectroscopy and diffraction/scattering, future investigations utilizing XFELs would be able to reveal the detailed dynamics of both the electronic and ionic systems.

## Method

### DFT-MD and XANES calculations of high temperature liquid Cu

In order to determine the electron temperature from the XANES measurement, first principle density functional theory - molecular dynamic (DFT-MD) simulations and ab-initio derived dipole-matrix elements calculation for liquid copper at the ionic temperature of 3800 K were performed. The atomic configurations for Cu were generated first principles using the quantum-espresso package^35^. A supercell containing 32 Cu atoms was used, and several picoseconds of MD simulations were performed after the equilibration process. A plane wave expansion was performed with E_cut_ = 40 Ry, and an ultrasoft pseudopotential was used to describe the electron-ion interaction. The Perdew-Burke-Ernzerhof generalized gradient approximation (PBEGGA) exchange correlation functional was used to approximate the many-body exchange correlation effect.

For selected uncorrelated atomic configurations, dipole matrix elements were calculated using the prescription described in ref. 36. An average was taken over the matrix elements for different Cu atoms in the supercell. As was discussed in the previous study, the profile of Cu L-edge dipole-matrix is not very sensitive to the ionic temperature in the range of this work, reflecting the metallic bonding of copper. However, the XANES, which is a product of dipole matrix and the Fermi distribution function, is sensitive to the electron temperature near the absorption edge reflecting the change of the Fermi distribution. In particular, at higher *T*_*e*_ (>1 eV), where electrons in d-bands are excited (partially unoccupied), x-ray excitations of the Cu 2p electrons into the d-bands become possible, leading to the development of a sharp absorption peak near the edge.

## Additional Information

**How to cite this article**: Cho, B. I. *et al.* Measurement of Electron-Ion Relaxation in Warm Dense Copper. *Sci. Rep.* **6**, 18843; doi: 10.1038/srep18843 (2016).

## Figures and Tables

**Table 1:** Summary of initial electron temperature right after the energy deposition, final sample temperature after equilibrium is achieved, and the characteristic relaxation time.

**Deposited energy density, MJ/kg**	**Initial electron temperature, (a + b) 10^4^ K**	**Final electron and ion temp, (b) 10^4^ K**	**Characteristic relaxation time, (τ) ps**
3.5	1.51 ± 1.0	0.64 ± 0.8	2.8 ± 0.4
5.3	1.98 ± 1.1	1.19 ± 1.0	3.4 ± 0.7
6.5	2.30 ± 1.5	1.30 ± 1.5	3.3 ± 1.0

Each value is determined from the fit of experimental data in the Fig. 2(e) using a causal exponential function (0, *t* < 0 and *a* exp(–*t*/*τ*) + *b*) convolved with the detector response.
